# The possibility of using xenogeneic phagocytes in wound treatment

**DOI:** 10.1371/journal.pone.0263256

**Published:** 2022-01-31

**Authors:** Andrey Yakovlev, Dmitry Tulin, Anna Savva, Anastasia Kruglikova

**Affiliations:** Laboratory of Insect Biopharmacology and Immunology, Saint-Petersburg University, Saint Petersburg, Russia; Lobachevsky University, RUSSIAN FEDERATION

## Abstract

Metamorphosis in the insect larva is associated with disintegration, engulf and digestion of larval tissues. These processes are accompanied by a significant shift in physiological parameters like high activity of hydrolytic enzymes and decrease of pH. In the way, the metamorphosing larva resembles the processes occurring in the wound at the stage of inflammation. Based on this thesis, we put forward the idea of the possibility of using insect phagocytes in the wound treatment. The search for a suitable insect cell line and the study of its properties were the purpose of the work. The abilities of insect phagocytes to retain viability and functional activity under conditions physiological for humans were also investigated. We found that blue blowfly *Calliphora vicina* larvae had histolysocytes, a specialized population of professional phagocytes involved in the histolysis. *In vitro*, histolysocytes possess high phagocytic activity to fragments of vertebrate soft tissues and debris. These cells retain viability and functional activity for a long time under conditions that are physiological for vertebrate cells. Moreover histolysocytes can realize the humoral control over the bacteria through the synthesis of antimicrobial peptides. So histolysocytes have the potential to be used as xenogeneic phagocytes in the wound treatment. The data obtained allow proceeding to experiments on laboratory animals for studying the effect of such therapy on the wound healing process.

## Introduction

Research on metazoan responses to injury was initiated as early as the end of the 19th century when Ilya Mechnikov performed his famous experiment with a starfish larva and a thorn from a tangerine tree [1]. Further studies have shown that the reaction to tissue damage in different animals, starting from primitive forms, had a stereotypical pattern and manifested itself in the migration of mobile cellular elements into the alteration zone with the subsequent isolation of the latter. This process is the initial and decisive stage for the onset of an inflammation response [2].

In vertebrates, phagocyte migration is preceded by the reaction of the capillary section of a well-developed vascular system. It is the reaction of the vessels (stasis, permeability hanging) along the border of the alteration zone that isolates the site of inflammation preventing the spread of microorganisms and the toxic products of necrobiosis throughout the body. In higher vertebrates, such a reaction acquires an additional biological meaning associated with the presence of adaptive immunity and aimed at excluding the possibility of entry of tissue deconstruction proteins (autoantigens) from the inflammation zone to the lymphatic system with the subsequent development of an immune response to them [3]. At this stage, any presence of cells of adaptive immunity is excluded in the emerging alteration zone, as well as their involvement in acute (non-chronic) inflammation. The processes of inflammation develop owing to low-specificity mechanisms of innate immunity, and the central role at this stage is played by phagocytes [4, 5].

In humans, a deficit of phagocytes in the wound or a decrease in their activity causes a complication of the wound healing process and contributes, among other factors, to the development of post-surgery sepsis [6]. There are no effective methods of substitution therapy in the case of phagocyte failure with the exception of bone marrow transplantation [7]. The use of humans as phagocyte donors is associated with high economic costs and biosafety problems. As a potentially powerful alternative, xenogeneic (non-human, non-mammalian and even invertebrate) cells theoretically could be used in wound treatment.

Although xenotransplantation representing the transplantation of living cells, tissues or organs from one species to another, is viewed as a promising method for regenerative medicine [8], there currently is an absence of practical results in this area. The main reasons are the unsolved histocompatibility problem which resulting from the risk of the development of undesirable reactions (allergies, rejection, etc.) and the problem of the transmission of infection with xenogeneic cells or tissues [8–10]. However, the presence of wound-specific conditions associated with the formation of a barrier along the viable tissue border makes it possible to utilize various heterogeneous materials in wound treatment, e.g., sorbents, biopolymers, and heterologous enzyme medications [11]. The spread of xenogeneic cells and the debris beyond the alteration zone also might be excluded.

Inflammation processes in the wound remarkably shift the physiological parameters from normal values. Necrobiotic toxins, lysosomal and hydrolytic enzymes, as well as increased acidity, create a hostile environment [12]. In this regard, a metamorphosing insect larva can be viewed as a model for a wound. In a larva, all tissues undergo histolysis and subsequent reutilization, similar to the processes taking place in a wound, and the parameters of the internal environment are also similarly shifted to a significant degree from baseline physiological values [13]. For example, the activity of hydrolytic enzymes in the hemolymph is high [14, 15] and the pH is changed dramatically. A pattern in the change in acidity [16, 17] correlates especially precisely with that of a wound in the inflammation phase in the sense that low pH values give way to higher ones [18]. And it is phagocytes circulating in the hemolymph that are stay alive and carry out the histolysis and tissue remodeling programs [19]. In addition, there exists a well-documented evolutionary conservatism of certain regulatory and effector mechanisms of innate immunity at the molecular level in insects and mammals [20–22].

The aim of this work was to search and collect suitable insect phagocytes, as well as a subsequent study of their activity *in vitro* to assess the possibility of further practical use in the substitution therapy of the phagocyte deficiency states.

Blowfly “surgical larvae” having been used over the centuries in wound treatment [23, 24] were looked at as a potential source of xenogeneic phagocytes. The blowflies were chosen due to their biomass high growth rate [25], the well-developed technology of large-scale cultivation [26, 27] and the possibility of insect culture sterilization [28, 29]–prerequisites for the target cell collecting and use.

## Materials and methods

### Insects

Experiments were performed with a laboratory strain of blue blowfly *Calliphora vicina* R.-D. (Diptera: Calliphoridae) originating from St. Petersburg area (North-West Russia) and characterized by stable larval diapause [30]. To synchronize development, a laboratory culture of *C*. *vicina* larvae was prompted to enter diapause by placing at +6°C after gut emptying. The diapausing larvae were transferred to +24°C so that they could resume development when required.

### Hemocyte collection and cultivation

Hemolymph was obtained from previously sterilized in 70% ethanol larvae by puncture of the integument of the head segment area. Hemolymph was collected directly in the ice-cold 0.05 M Dulbecco’s phosphate-buffered saline (DPBS, BioloT, Russian Federation). The cells were precipitated by centrifugation at 100 × g for 5 minutes. The supernatant (plasma) was removed, the cells were resuspended, and the procedure was repeated. The obtained hemocytes were transferred to the culture medium–DPBS with 20% diapause larva’s heat-inactivated plasma (cell-free plasma was heated up to +60°C for 30 minutes; denatured protein was excluded via centrifugation at 10,000 × g for 15 minutes) and a mixture of antibiotics (Sigma-Aldrich, St. Louis, MO, USA). For subsequent analysis, the cells in the culture medium were placed on a slide (Deltalab, Spain, Ref. D100003,) or a cell dish (Eppendorf AG, Germany, Cat. no.: 0030700112) until forming a monolayer (detected microscopically).

### Cytochemical reactions

#### Azure-eosin

The hemocyte monolayer was fixed in a 4% paraformaldehyde (Thermo Fisher Scientific, Frederick, MD, USA) in DPBS for 15 minutes, washed three times with DPBS, and regressively stained [31] with a 0.1% aqueous solution of azure (Sigma-Aldrich, St. Louis, MO, USA) and 0.1% aqueous solution of eosin (Sigma-Aldrich, St. Louis, MO, USA). The preparations were analyzed with a microscope Leica DMI 2500 (Leica Microsystems, Germany) in a bright field.

#### Nile blue

Identification of lysosomes was carried out according to the method by Lin et al. [32]. After the 5-minute incubation of a live hemocyte monolayer with a 0.025% solution of Nile blue (NevaReaktiv, Russia) in the culture medium, the preparation was washed three times with DPBS and analyzed with a microscope Leica DMI 2500 by the Differential interference contrast (DIC) / Nomarski methods.

#### Acridine orange

A monolayer of live hemocytes was incubated with a 0.002% solution of acridine orange (NevaReaktiv, Russia) in the culture medium for 5 minutes, washed with DPBS, and analyzed using a high-performance fluorescence of microscope Leica DMI 2500 (525 nm).

#### Acid phosphatase

Acid phosphatase activity in hemocytes was detected by the Gomori method modified by Chayen et al. [33]. Preliminary fixed in 4% paraformaldehyde solution hemocyte monolayer was transferred to the warmed staining rack and exposed simultaneously to the Gomori medium (solution A, 50 mM acetate buffer, pH 5.0, containing 0,132 g of lead nitrate (Sigma-Aldrich, St. Louis, MO, USA), and solution B, 3% sodium-β-glycerophosphate (Sigma-Aldrich, St. Louis, MO, USA) in distilled water, mixed carefully and filtered) for 24 h incubation at a temperature +37°C. The preparation was washed three times with DPBS and analyzed microscopically.

### Hemocyte interaction with extracellular matrix proteins

A suspension of larvae hemocytes in the culture medium was placed on fibronectin coated coverslips (Neuvitro Corporation, Camas, WA, USA, Cat. no.: GG-12-Fifronectin), collagen coated coverslips (Neuvitro Corporation, Camas, WA, USA, Cat. no.: GG-12-Collagen), or laminin coated coverslips (Neuvitro Corporation, Camas, WA, USA, Cat. no.: GG-12-Laminin). Hemocyte spreading was studied in 30 minutes after washing the preparation with DPBS.

### Actin cytoskeleton staining

To observe the actin cytoskeleton, hemocytes placed on extracellular matrix (ECM) proteins were stained with phalloidin-Atto665 (Sigma-Aldrich, St. Louis, MO, USA). Before staining the hemocyte monolayer was fixed in a 4% paraformaldehyde in DPBS for 10 minutes, washed in DPBS twice, treated with 0,1% Triton X 100 PRS (Panreac, Spain) in DPBS for 3 minutes, and, after washing, incubated in a 1% bovine serum albumin (Sigma-Aldrich, St. Louis, MO, USA) for 25 minutes. The staining with a phalloidin-conjugate working solution took 25 minutes. After washing in DPBS, the preparation was added with anti-fade protector ProLong Gold Antifade Mountant (Thermo Fisher Scientific, Frederick, MD, USA). The preparation was analyzed using fluorescence microscope (665 nm).

### Transmission electron microscopy (TEM)

Hemocytes were fixed in a mixture of 2% paraformaldehyde and 2.5% glutaraldehyde (Thermo Fisher Scientific, Frederick, MD, USA) in a culture medium. Postfixation was carried out with 1% osmium tetroxide (Sigma-Aldrich, St. Louis, MO, USA) in 0.05 M cacodylate buffer (Vekton, Russian Federation), pH 7.4, using a low-temperature automatic water replacement system Leica EM AFS2 (Leica Microsystems, Germany).

The samples were infiltrated with epoxy resin (Agar Scientific Ltd, UK) / acetone (Vekton, Russian Federation) (1:3) for 30 min, followed by resin / acetone (1:2) for 30 min, followed by 1 h with resin / acetone (1:1) under room temperature. The samples were then transferred into the 100% resin; the polymerization was carried out over 24 h at +60°C. Resin blocks were carefully trimmed using a Leica EM UC7 trimmer. Ultrathin slices were collected on the mesh copper grids coated by carbon film (SPI Supplies, West Chester, PA, USA), post-stained with 1% uranyl acetate (Agar Scientific Ltd, UK) in water for 5 min, and with a lead solution for 7 min, and washed in distilled water. The material was then analyzed with a transmission electron microscope JEM– 1400 (Jeol, Japan).

### Cytofluorimetric analysis

Hemolymph from 10 larvae was collected in cold DPBS. A 4’,6-diamidino-2-phenylindole (DAPI) solution (Sigma-Aldrich, St. Louis, MO, USA) was added to the suspension. The positive peak of DAPI was analyzed in BD FACSAria III with the parameters FSC (cell size) and SSC (granularity). A total of 50,000 events were collected.

### The detection of antimicrobials released by hemocytes

Hemocytes from 15 prepupae were incubated in DPBS supplemented with 20% diapause larva’s heat-inactivated plasma and antibiotics for 18 hours at a temperature of +22°C. Then, the culture medium was collected, acidified with 0.1% trifluoroacetic acid (TFA, Sigma-Aldrich, St. Louis, MO, USA) to a final concentration of 0.05%, and applied to a prepared reversed-phase SepPak C18 cartridge (Waters Corporation, Milford, MA, USA) for fractionating. Highly hydrophilic compounds were removed by cartridge washing with 0.05% TFA. Hydrophobic compounds were eluted with 50% acetonitrile (Cryochrom, Russian Federation) in 0.05% TFA and lyophilized. For further high-performance liquid chromatography fractionation, the lyophilizate was dissolved in deionized water and totally applied to a Vydac C18 column (250 × 10 mm, 5 μm, Grace, Columbia, MD, USA), equilibrated with 0.05% TFA. Substances were eluted with a linear gradient of acetonitrile from 2 to 50% for 50 minutes. Chromatographic fractions automatically collected with 1-min intervals were lyophilized and dissolved in 25 μl of deionized water. The antimicrobial components in the fractions were detected by a plate-growth inhibition assay [34], examining the size of the zones of complete growth inhibition of *Micrococcus luteus* A270 or *Escherichia coli* D31 around 9 μl fraction aliquots. Chromatographic fractions of the hemocyte culture with the peak antimicrobial activities were tested again in three replicates and compared with corresponding fractions of free-cell cultural medium (control).

### *In vitro* model of a wound

Muscle fiber bunches were obtained by mechanical pinching from a piece of fresh chicken / pig meat. A suspension of myofibrils obtained by grinding of fresh chicken / pig muscle fibers and subsequent destruction by pipetting was added to the hemocyte monolayer in a cell culture dish (Eppendorf AG, Germany, Cat. no.:0030700112).Then, the behavior of hemocytes was observed.

A suspension of muscle fibers / myofibrils was mixed with a suspension of hemocytes and the behavior of the cells including their behavior in a mixture of Gram-positive and Gram-negative bacteria was observed using an inverted microscope Nikon Eclipse TS 100 (Nikon, Japan) equipped with a time lapse camera.

To simulate the behavior of hemocytes on the wound surface, large (up to 3 cm) pieces of pig meat being immersed in a culture medium were covered with a suspension of *C*. *vicina* prepupal cells. Observation of hemocytes was carried out using light microscope or stereomicroscope equipped with time lapse camera.

Each of these experiments was repeated at least five times.

### Statistical analyses

The results are expressed as a mean ± SE. Continuous variables were compared by the non-parametric Mann-Whitney test in the Statistica 7.1 program (StaSoft Inc., USA).

## Results

### *C*. *vicina* hemocyte characteristics

**Fig 1:**
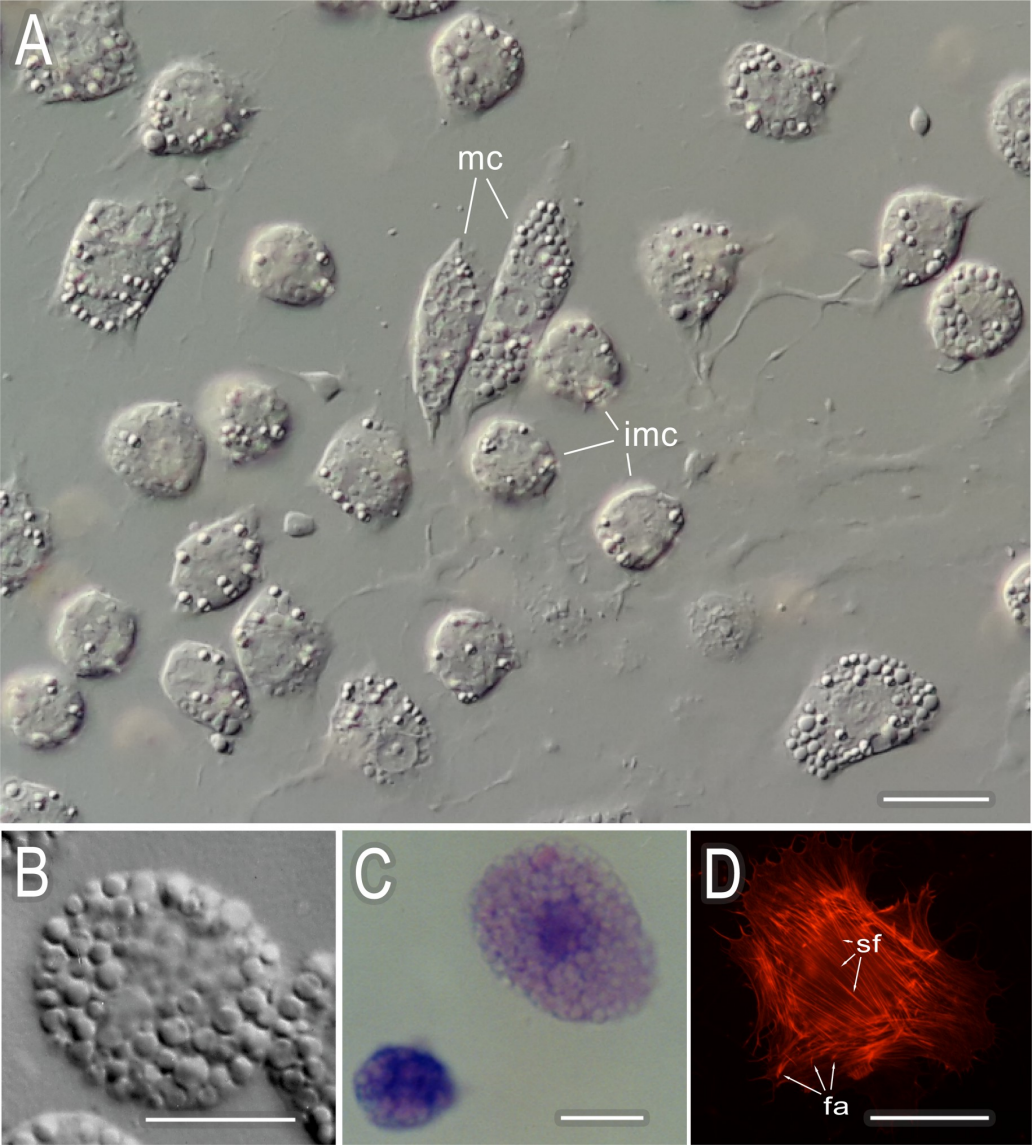
Differentiation of *C*. *vicina* phagocytes. A–live hemocytes of the diapausing larva. The heterogeneity of the population is apparent: immature cells, imc (smaller, rounded, and containing few inclusions) and mature cells, mc (bigger, sprawled, and full of inclusions) are present in circulation at the same time; slightly differentiated forms prevail. Nomarski optics. B–intact mature pupal phagocyte: large cell, the entire cytoplasm is filled with specific inclusions. Nomarski optics. C–diapausing larva live hemocytes of the stained with azure-eosin: the cytoplasm of a slightly differentiated phagocyte shows strong basophilic staining with azure (blue) due to a large amount of RNA present in young cells. Inclusions filling the mature cell are stained with protein-dye eosin (pink). Bright field. D–differentiated prepupal hemocyte on collagen. The cell is conspicuously spread out and forms numerous focal adhesions (fa) and actin stress fibers (sf). Fluorescence microscopy. Scale bar– 10 μm.

The morphological diversity of cells was observed in temporary mounts of hemolymph of post-diapause *C*. *vicina* larvae (i.e., larvae that were committed to pupariation). Small round cells with single granule-like inclusions and larger cells with the same morphological features were shown to be present simultaneously in circulation (Fig 1A and 1B). The hemocyte morphotypes observed could be ranked by the degree of expression of the same morphological characters.

Large, living hemocytes of the larva were able to spread out on the slide while small cells retained their shape for a long period of time. When stained with azure and eosin (Fig 1C), the cytoplasm of small hemocytes exhibited stronger basophilic staining, which could be explained by the presence of a large amount of RNA in the cytoplasm and was indicative of the weak differentiation of these cells. There were granule-like inclusions that completely filled the cytoplasm of large hemocytes and were well stained with eosin indicating the proteinaceous nature of these structures. These hemocytes clearly represented mature forms, and the above-mentioned series reflects the consecutive stages of differentiation of the same cell line. Mature hemocytes spread out well on slides coated with ECM proteins. Cytoskeletal protein staining (Fig 1D) revealed that such hemocytes formed numerous focal adhesions and stress fibers, suggesting the presence of receptors for ECM components on these cells.

**Fig 2:**
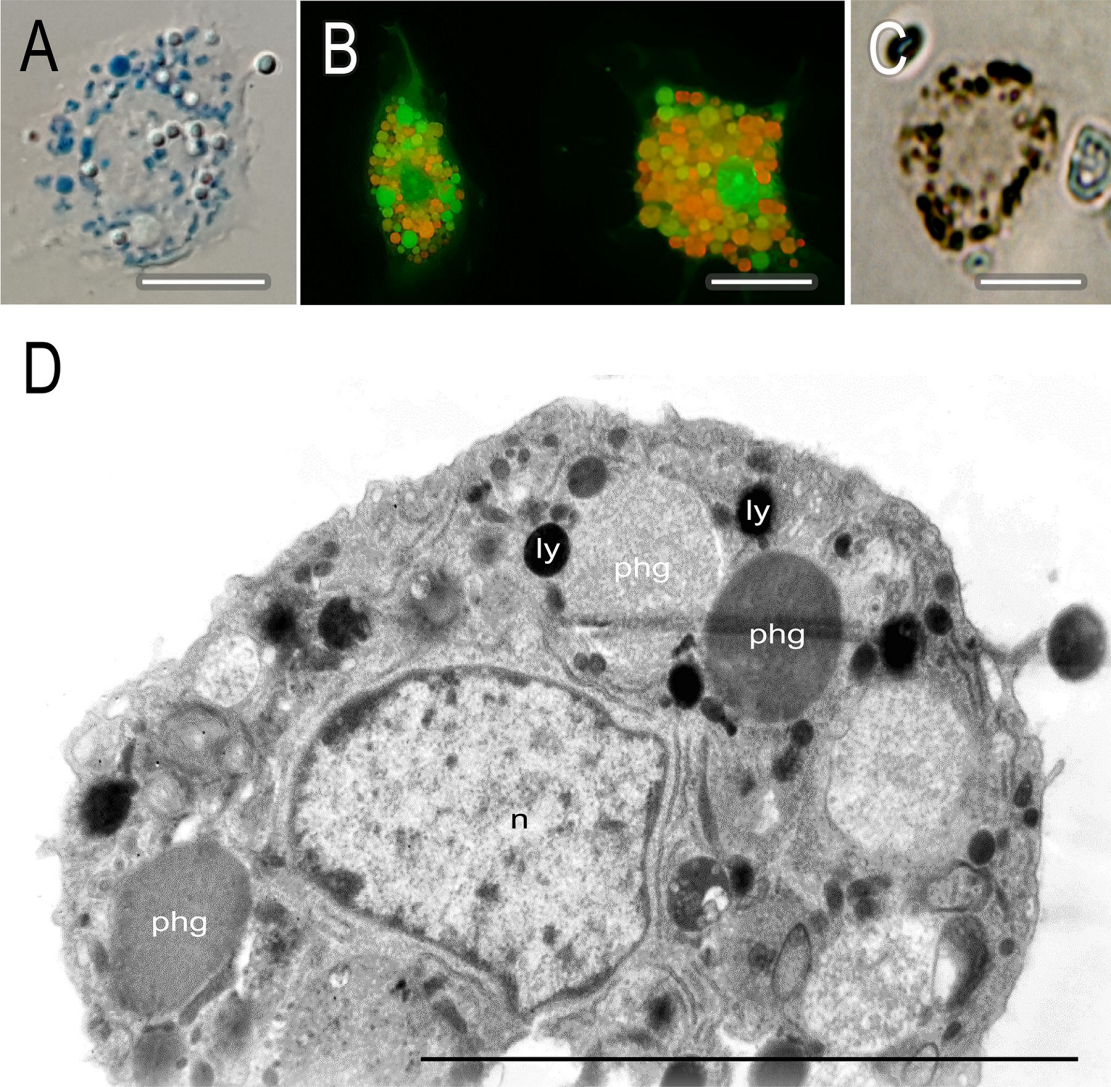
Cytoplasmic inclusions of *C*. *vicina* phagocytes. A–live hemocytes in a *C*. *vicina* larva. Blue grain–lysosomes: Nile blue. Nomarski optics. B–live prepupal hemocytes with 0.002% acridine orange. Granule-like inclusions have a different fluorescence color, which confirms their catabolic nature. Fluorescence microscopy. C–prepupal hemocyte, reaction to acid phosphatase. Brown pigment–acid phosphatase activity zones. Bright field. D–the fine structure of a differentiated prepupal hemocyte. The heterogeneity of the catabolic hemocyte inclusions is clearly visible. Ly–lysosomes, phg–phagosomes at different stages of maturation (from the light to the dark), n–nucleus. TEM. Scale bar– 10 μm.

To determine the nature of the cytoplasmic inclusions in circulating hemocytes of *C*. *vicina* larva and pupa, a number of cytochemical reactions were carried out and ultrastructure level morphology was studied (Fig 2). Tests with Nile blue revealed the presence of numerous lysosomes in the cytoplasm of hemocytes (Fig 2A). Incubation with acridine orange demonstrated a pronounced heterogeneity in the fluorescence of granule-like inclusions (Fig 2B) that was not typical of true granulocytes. The fluorescence spectrum of acridine orange was determined by the degree of polymerization of the stain. The latter depends solely on the pH of the medium: a decrease in pH shifts the fluorescence color from green to red [35]. Thus, fluorescence heterogeneity confirms the catabolic nature of hemocyte inclusions. Moreover, some fluorescence colors of stain polymers (green-yellow-orange-red-burgundy) probably reflect the consecutive stages of maturation of phagolysosomes. Reaction to acid phosphatase, a lysosome marker enzyme, indicated the presence of enzyme activity in cytoplasmic inclusions (Fig 2C). The examination of hemocytes with TEM confirmed the presence of lysosomes and growing phagolysosomes in the cytoplasm (Fig 2D).

**Fig 3:**
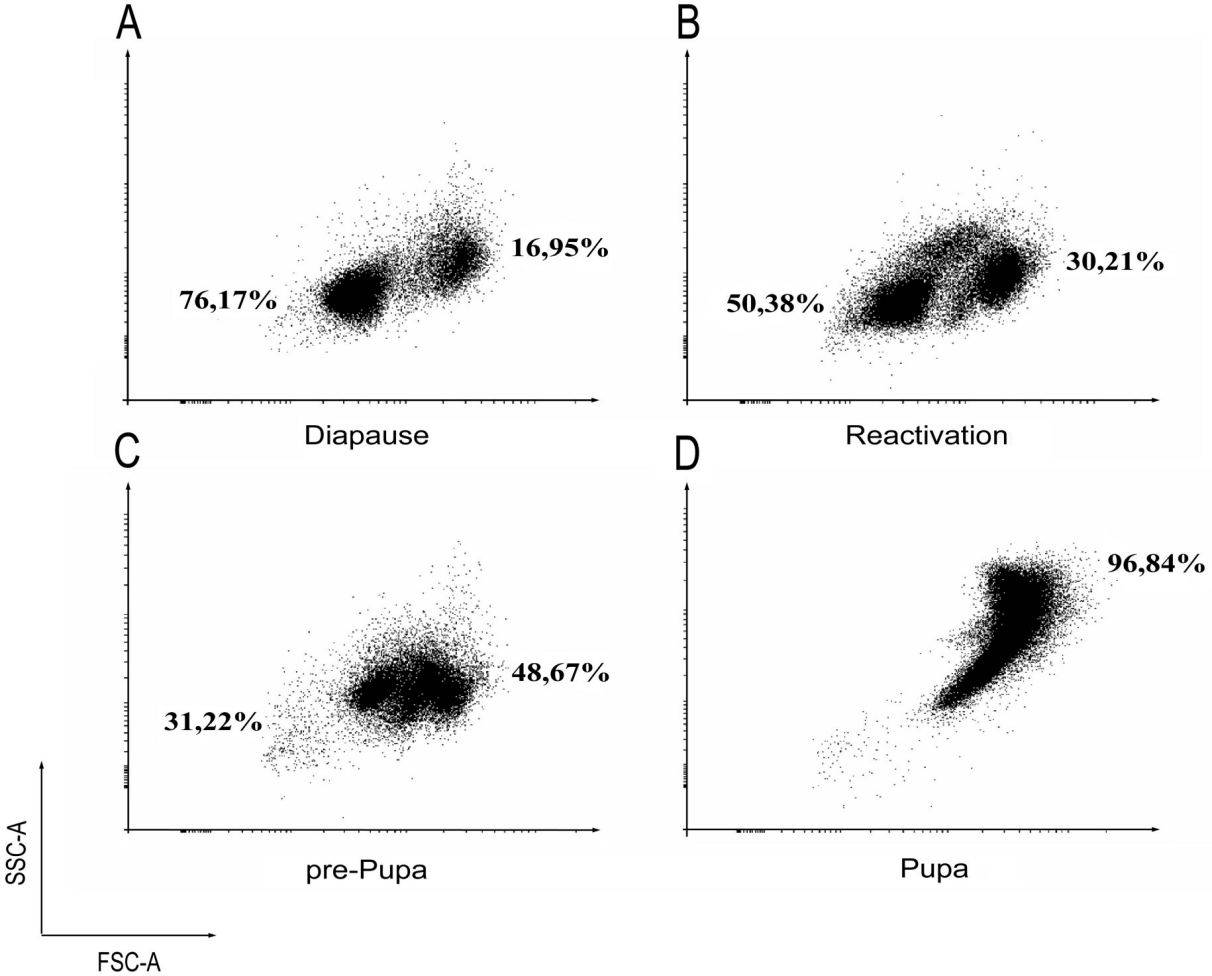
The dynamics of the population structure of *C*. *vicina* circulating hemocytes. As one progresses to the pupal stage, the cell migration from the left population to the right is observed that indicates the cell differentiation process. Cytofluorimetric analysis data.

Cytofluorimetric analysis (Fig 3) showed that the hemocytes of a diapausing larva formed two clearly defined populations: less differentiated hemocytes and more differentiated ones, the latter showing a larger size and greater granularity (left and right aggregations, respectively, in the cytogram 3A). As the larva approached pupariation, the cells migrated from one population to the other. The migration reflects the hemocyte differentiation. At the prepupal stage, populations almost merged. At the same time, the maturation of differentiated cells continued: in particular, the size increased (FSC-A mean from 9179 (3B) to 12,540 (3C)) and so did granularity (SSC-A mean from 794 (3B) to 1740 (3C)). After the beginning of tissue histolysis (pupa formation, 3D), the size (FSC-A mean– 36122) and granularity (SSC-A mean– 8019) of circulating hemocytes increased sharply, which indicates the completion of cell maturation.

**Fig 4:**
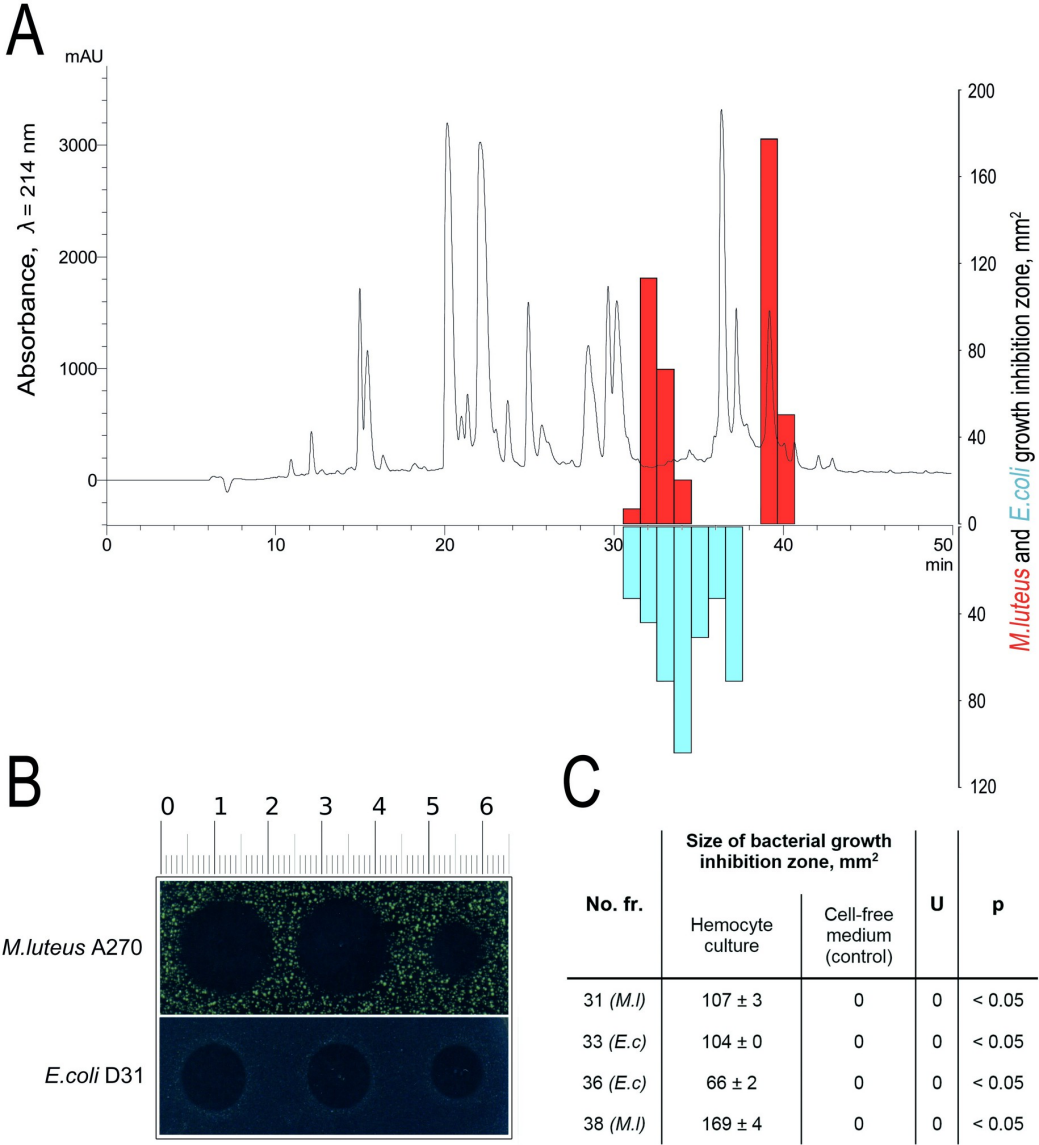
Antimicrobial components secreted by prepupal hemocytes of *C*. *vicina*. The hemocyte culture medium was fractionated chromatographically (A) and fractions were tested by the plate-growth inhibition method (B) for the presence of antimicrobial components. The areas of zones of complete growth inhibition of Gram-positive *M*. *luteus* A270 (above the X-axis) and Gram-negative *E*. *coli* D31 (below the X-axis) were mapped on the chromatographic profile; antimicrobial activity in the represented fractions was absent in the control. Chromatographic fractions with the peak antimicrobial activities were re-tested in three replicates and compared with the control by the non-parametric Mann-Whitney test (C).

The medium after prepupal hemocyte incubation was examined for the possible presence of antimicrobial factors secreted by the cells. After testing chromatographic fractions with the plate-growth inhibition method (Fig 4), it was found that anti-Gram-positive activity was confined to fractions 30–33 (peaking in fraction 31) and 38–39 (peaking in fraction 38), and anti-Gram-negative activity was confined to fractions 30–34 (peaking in fraction 33) and 35–36 (peaking in fraction 36). In the control, the corresponding fractions did not contain any antimicrobial components. Chromatographic mobility, i.e., the distribution pattern of antimicrobial activity and elution time, corresponded to that in *C*. *vicina* larval antimicrobial peptides (AMP) have described previously by Chernysh et al. [36].

### Hemocyte activity in the wound model

**Fig 5:**
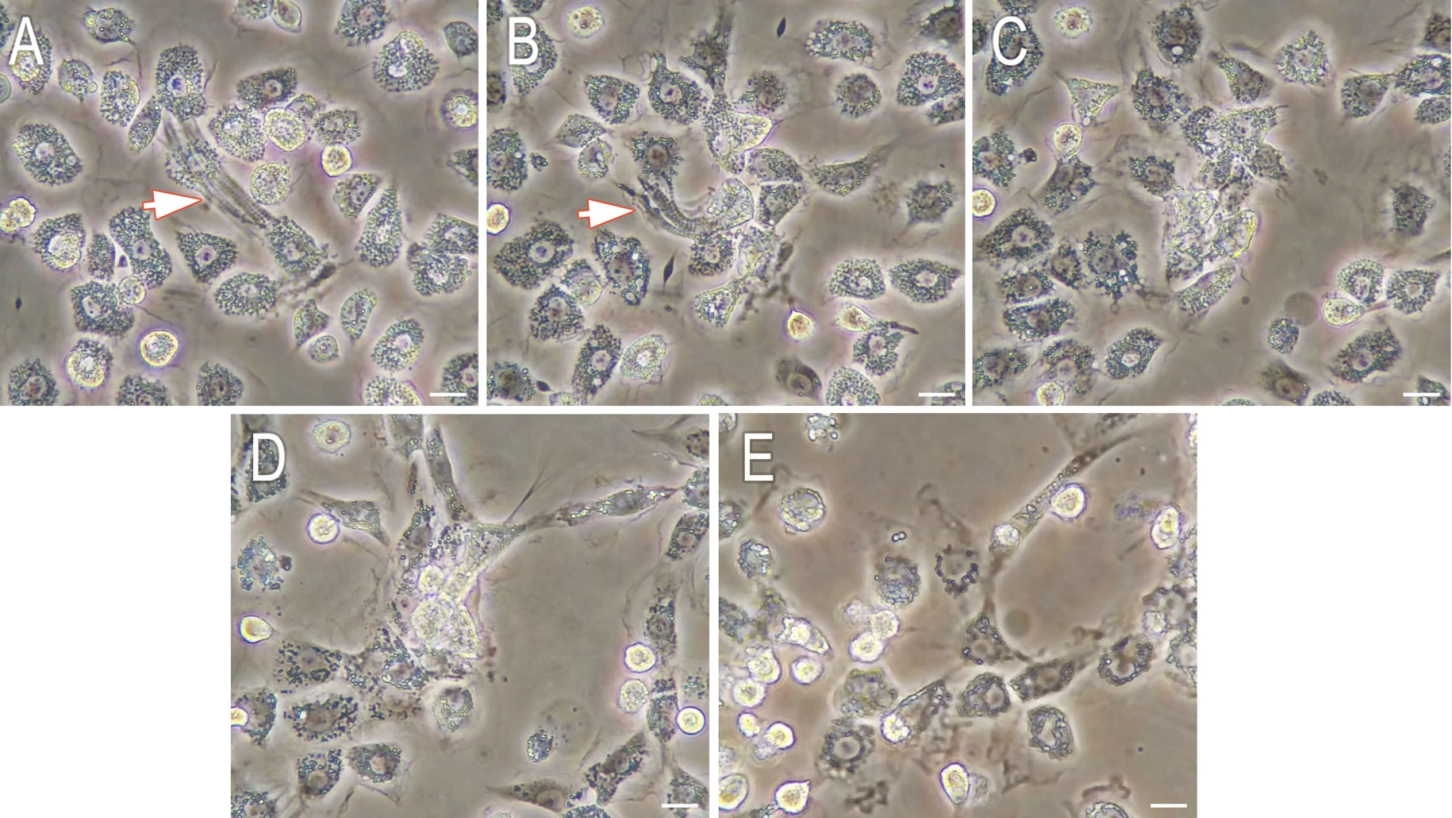
Phagocytosis of pig myofibrils by the hemocytes of a *C*. *vicina* prepupa. Starting to contact with the muscle fiber (arrow) after 30 minutes of cultivation (A), hemocytes disassemble and engulf it gradually (B—2 h 30 min, C—3 h 30 min, D—5 h 30 min of cultivation) up to complete phagocytosis by 24 hours (E). Scale bar– 10 μm.

To assess (simulate) the behavior of *C*. *vicina* hemocytes in a wound, an enriched population of prepupal phagocytes was incubated in a medium containing cell debris and fragments of vertebrate (chicken, pig) muscle tissue. Hemocytes collected from larvae at the onset of histolysis completely disassembled and phagocytosed even large muscle fibers that much more exceed hemocyte size (Fig 5; the whole process is available on the video file in S1 Video). During the engulfment of large muscle tissue fragments hemocyte cooperation was observed. As well phagocytes effectively absorbed and engulfed separate myofibrils (see S2 Video).

The analysis of the hemocytes behavior on the wound-like surface showed that prepupal phagocytes quickly attach to the substrate and actively move along it (see S3 Video). Under cultivation the cells were shown to form mobile clusters being a consequence of the cooperation mentioned above.

It should be noted that all of the simulation experiments were time-consuming (tens of hours) and were carried out under conditions that are physiological for the cells and tissues of vertebrates. This did not affect the viability and functional activity of hemocytes.

## Discussion

The possibility of using *C*. *vicina* larvae as a source of specific highly active phagocytes was investigated. This species was characterized by a deep diapause before the start of the tissue remodeling processes. Commitment to pupariation in *C*. *vicina* larvae is accompanied by a mass appearance of immature predecessors of a specialized line of phagocytes in the hemolymph. The ratio of these specialized phagocytes is more than 90% of all circulating hemocytes. In the wandering larva, the morphological diversity of hemocytes in circulation is a consequence of a simultaneous presence of different stages of cells of the same differentiation line. This agrees with the literature data on the absence of specialized hematopoietic organs in dipteran larvae. The differentiation of these cells occurs directly in the hemolymph and is manifested in the following: an increase in cell size, the formation of the lysosomal apparatus, the appearance of specific catabolic inclusions in the cytoplasm, the expression of receptors for the ECM elements, increased amoeboid movement, and the development of the cytoskeleton. All of these features characterize this cell line as a population of highly active professional phagocytes.

The changes observed in the structure of the circulating hemocyte population as the larva prepares for pupariation clearly indicate that these cells play a leading role in histolysis. The involvement of hemocytes in the digestion of disintegrated larval tissues was previously mentioned in the literature [37]. For this reason, the term “histolysocytes” was proposed. In the authors’ opinion, the use of this term is all the more justified as the phagocytic activity of these cells is uninduced. That is, this activity is not associated with the development of the immune reaction but has a normal physiological nature.

Based on the data above, the authors believe the prepupal hemocytes to be most promising in light of the aim of this study. First, during the prepupal stage, there are already no immature forms in circulation, and hemocytes are completing their functional differentiation. Second, the change in granularity and cell size observed during the transition to the pupal stage is a consequence of active phagocytosis of disintegrated larval tissue by the hemocytes. Therefore, in the authors’ opinion, it seems more promising to use “hungry phagocytes one step before” for the necrotic soft tissue engulfment.

It was discovered that, during cultivation, histolysocytes released substances into the medium that are toxic to Gram-positive and Gram-negative bacteria. It was previously found that the AMP complex of *C*. *vicina* larvae includes peptides that belong to defensin, cecropin, diptericin, and proline-rich peptide families. A useful feature of this complex is the inability of bacteria to evolve resistance to it [36]. Moreover, the AMP complex is also active against microorganisms that form biofilms [38].

The ability of hemocytes to condition the medium as they release antimicrobial factors results in the sustained phagocytic activity of these cells even after exposure to a mixture of Gram-positive and Gram-negative bacteria. At the same time, histolysocytes do not show phagocytic activity against microorganisms.

The simulation experiments that model the possible behavior of histolysocytes in a wound demonstrate: (a) the ability of histolysocytes to retain viability and functional activity for a long time under conditions that are not physiologically normal to them; (b) the ability to possess high phagocytic activity to fragments of vertebrate necrotic soft tissues and debris (in contrast to living cells of vertebrates–the data obtained on the tumor cells, not shown); (c) the presence of receptors for the ECM components on the membranes of these cells; (d) rapid amoeboid movement and a well-developed lysosomal apparatus; (e) the ability to cooperate in the clusters similar to vertebrate phagocytes [39, 40]; (f) control of the activity of microorganisms in the medium by synthesizing and releasing an array of antimicrobial peptides. In view of all these findings, the authors hope that histolysocytes could be successfully used in the early stages of the wound healing process (i.e., necrotic changes and the formation of an inflammation).

The development of a protocol for the use of xenogeneic cells to compensate for the deficiency of phagocytes in the wound should include: 1) the search for and true assessment of candidate cells; 2) assessment of the influence of xenogeneic phagocytes on the wound healing process in the laboratory animals; 3) further transition to the pilot of clinical trials in human. The current study is limited only with the first point, and its positive results allow moving on to experiments on animals and hoping for the successful implementation of the project as a whole.

## Supporting information

S1 Video In vitro disintegration and phagocytosis of large porcine muscle fibers (red arrow) by mature histolysocytes of the *C*. *vicina* larva. The size of fragment significantly exceeds the size of hemocytes. Insects cells exhibit the cooperation in the process of debris destroying. Scale bar—10μm. Phase-contrast microscopy, time-lapse, speed– 120x.
(MP4)

S2 Video In vitro utilization of separate myofibrils by histolysocytes of the *C*. *vicina* larva. Insect cells actively engulf muscle fragments. Scale bar—10μm. Phase-contrast microscopy, time-lapse, speed– 90x.
(MP4)

S3 Video *C*. *vicina* hemocytes on the substrate simulating the wound surface 3 hours after inoculation. Histolysocytes remain alive and keep functional activity under conditions that are physiological for vertebrate cells. Сlusters formed by hemocytes can be observed. Cluster appearance result from the hemocyte cooperation in the process of debris phagocytosis. Scale bar– 100 μm. Stereo microscope, time-lapse, speed– 90x.
(MP4)

## References

[1] Desowitz RS. Thorn in the starfish: the immune system and how it works. Desowitz RS, editor. W. W. Norton & Company; 1988. 276 p. Available from: https://www.wwnorton.co.uk/books/9780393305562-thorn-in-the-starfish

[2] Rowley AF, Ratcliffe NA. The granular cells of *Galleria mellonella* during clotting and phagocytic reactions *in vitro*. Tissue Cell. 1976 Jan;8(3):437–46. Available from: https://linkinghub.elsevier.com/retrieve/pii/0040816676900045 doi: 10.1016/0040-8166(76)90004-5 982421

[3] Goldsby RA, Kindt TJ, Osborne BA, Kuby J. Immunology. Fifth. Goldsby RA, editor. W.H. Freeman; 2003. 555 p.

[4] Huber-Lang M, Lambris JD, Ward PA. Innate immune responses to trauma review-article. Nat Immunol. 2018;19(4):327–41. Available from: 10.1038/s41590-018-0064-8 29507356 PMC6027646

[5] Inflammation Medzhitov R. 2010: New Adventures of an Old Flame. Cell. 2010;140(6):771–6. doi: 10.1016/j.cell.2010.03.006 20303867

[6] Andrews T, Sullivan KE. Infections in patients with inherited defects in phagocytic function. Clin Microbiol Rev. 2003;16(4):597–621. Available from: https://cmr.asm.org/content/16/4/597 doi: 10.1128/CMR.16.4.597-621.2003 14557288 PMC207096

[7] Watanabe C, Yajima S, Taguchi T, et al. Successful unrelated bone marrow transplantation for a patient with chronic granulomatous disease and associated resistant pneumonitis and Aspergillus osteomyelitis. Bone Marrow Transplant. 2001;28:83–87. doi: 10.1038/sj.bmt.1703086 11498749

[8] World Health Organization. Division of emerging and other communicable diseases surveillance and control. Xenotransplantation: Guidance on Infectious Disease Prevention and Management. World Health Organization, 1998. Available from: https://apps.who.int/iris/handle/10665/65511

[9] Fishman JA, Scobie L, Takeuchi Y. Xenotransplantation-associated infectious risk: a WHO consultation. Xenotransplantation. 2012;19(2):72–81. doi: 10.1111/j.1399-3089.2012.00693.x 22497509 PMC3768267

[10] Cascalho M, Platt JL. Challenges and potentials of xenotransplantation. Clinical Immunology. 2008;1215–1222. 10.1016/B978-0-323-04404-2.10081-8

[11] Shtilman MI. Polymeric Biomaterials. Part I. Polymer Implants (New Concepts in Polymer Science). VSP International Science; 2003. 305 p.

[12] Nagoba BS, Suryawanshi NM, Wadher B, Selkar S. Acidic environment and wound healing: A review. Wounds. 2015;27(1):5–11.

[13] Johnson CG. Insect Migration: Aspects of its physiology. In: The Physiology of Insecta. Elsevier; 1974. p. 279–334. Available from: https://linkinghub.elsevier.com/retrieve/pii/B9780125916035500115

[14] Rabossi A, Acion L, Quesada-Allue LA. Metamorphosis-associated proteolysis in *Ceratitis capitata*. Entomol Exp Appl. 2000 Jan;94(1):57–65. Available from: http://doi.wiley.com/10.1046/j.1570-7458.2000.00604.x

[15] Smith E, Birt LM. Proteolytic activity during the metamorphosis of the blowfly Lucilia. Insect Biochem. 1972 Jun;2(6):218–25. Available from: https://linkinghub.elsevier.com/retrieve/pii/002017907290056X

[16] Histolysis Agrell I., histogenesis, and differentiation during insect metamorphosis in relation to metabolic changes. Development. 1953;1(3):279–82.

[17] Armbruster L, Levy M, Mathieu MN, Bautz AM. Acid phosphatase activity in the hemolymph, hemocytes, fat body and salivary glands during larval and prepupal development in *Calliphora erythrocephala* (Diptera: Calliphoridae). Comp Biochem Physiol Part B Comp Biochem. 1986 Jan;84(3):349–54. Available from: https://linkinghub.elsevier.com/retrieve/pii/030504918690088X

[18] Schneider LA, Korber A, Grabbe S, Dissemond J. Influence of pH on wound-healing: a new perspective for wound-therapy? Arch Dermatol Res. 2007 Jan 16;298(9):413–20. Available from: http://link.springer.com/10.1007/s00403-006-0713-x 17091276 10.1007/s00403-006-0713-x

[19] Hori S, Kobayashi A, Natori S. A novel hemocyte-specific membrane protein of Sarcophaga (flesh fly). European Journal of Biochemistry. 2000;267:5397–5403. Available from: doi: 10.1046/j.1432-1327.2000.01578.x 10951197

[20] Browne N, Heelan M, Kavanagh K. An analysis of the structural and functional similarities of insect hemocytes and mammalian phagocytes. Virulence. 2013 Oct 27;4(7):597–603. Available from: http://www.tandfonline.com/doi/abs/10.4161/viru.25906 23921374 10.4161/viru.25906 PMC3906293

[21] Kavanagh K, Reeves EP. Exploiting the potential of insects for in vivo pathogenicity testing of microbial pathogens. FEMS Microbiol Rev. 2004 Feb;28(1):101–12. Available from: https://academic.oup.com/femsre/article-lookup/doi/10.1016/j.femsre.2003.09.002 14975532 10.1016/j.femsre.2003.09.002

[22] Rämet M, Pearson A, Manfruelli P, Li X, Koziel H, Göbel V, et al. Drosophila scavenger receptor CI is a pattern recognition receptor for bacteria. Immunity. 2001 Dec;15(6):1027–38. Available from: https://linkinghub.elsevier.com/retrieve/pii/S1074761301002497 doi: 10.1016/s1074-7613(01)00249-7 11754822

[23] Kruglikova A, Chernysh S. Surgical maggots and the history of their medical use. Entomological Review. (2012). 93. 667–674. 10.1134/S0013873813060018.

[24] Naik G, Harding KG. Maggot debridement therapy: the current perspectives. Chronic Wound Care Management and Research. 2017;4:121–128. Available from: 10.2147/CWCMR.S117271

[25] Donovan SE, Hall MJR, Turner BD, Moncrieff CB. Larval growth rates of the blowfly, *Calliphora vicina*, over a range of temperatures. Medical and Veterinary Entomology. 2006;20:106–114. Available from: 10.1111/j.1365-2915.2006.00600.x 16608495

[26] Sherman RA, Wyle FA. Low-cost, low-maintenance rearing of maggots in hospitals, clinics, and schools. The American Journal of Tropical Medicine and Hygien 1996;54(1):38–41. Available from: 10.4269/ajtmh.1996.54.38 8651366

[27] Nadtochii L, Baranenko D, Melchakov R, Muradova M, Istomin A, Istomin A. Investigation of fly larvae *Lucilia caesar* application in pet feed composition. Agronomy Research. 2019;17(6):2359–2372. Available from: 10.15159/AR.19.209

[28] Mohd Masri S, Nazni WA, Lee HL, Rogayah T, Subramaniam S. Sterilisation of *Lucilia cuprina* Wiedemann maggots used in therapy of intractable wounds. Tropical biomedicine. 2006;22:185–9. 16883286

[29] Gasz NE, Harvey ML. A new method for the production of sterile colonies of *Lucilia sericata*. Medical and Veterinary Entomology. 2017;(3):299–305. Available from: 10.1111/mve.12232 28402593

[30] Nesin AP, Simonenko NP, Numata H, Chernysh SI. Effects of photoperiod and parental age on the maternal induction of larval diapause in the blowfly, *Calliphora vicina*. Appl Entomol Zool. 1994;30(2):351–6. Available from: http://www.mendeley.com/research/geology-volcanic-history-eruptive-style-yakedake-volcano-group-central-japan/

[31] Mulisch M., Welsch U. (2015) Romeis—Mikroskopische Technik. Available from: 10.1007/978-3-642-55190-1

[32] Lin CW, Shulok JR, Kirley SD, Cincotta L, Foley JW. Lysosomal localization and mechanism of uptake of Nile blue photosensitizers in tumor cells. Cancer Res. 1991 May 15;51(10):2710–9. Available from: http://www.ncbi.nlm.nih.gov/pubmed/2021950 2021950

[33] Chaen J, Bitensky L, Butcher RG, Poulter LW. A Guide to Practica ln Histochemistry. Edinburgh: Oliver and Boyd, 1969.

[34] Chernysh SI, Gordya NA, Simonenko NP. Diapause and immune response: induction of antimicrobial peptides synthesis in the blowfly, *Calliphora vicina* R.-D. (Diptera, Calliphoridae). Entomol Sci. 2003;3:139–144.

[35] Wissing F, Sanchez CP, Rohrbach P, Ricken S, Lanzer M. Illumination of the Malaria Parasite *Plasmodium falciparum* Alters Intracellular pH. J Biol Chem. 2002 Oct;277(40):37747–55. Available from: https://linkinghub.elsevier.com/retrieve/pii/S0021925818365037 doi: 10.1074/jbc.M204845200 12140286

[36] Chernysh S, Gordya N, Suborova T. Insect antimicrobial peptide complexes crevent resistance development in bacteria. Cloeckaert A, editor. PLoS One. 2015 Jul 15;10(7):e0130788. Available from: https://dx.plos.org/10.1371/journal.pone.0130788 26177023 10.1371/journal.pone.0130788 PMC4503414

[37] Bautz A-M. Action des plasmatocytes sur divers tissus larvaires de *Calliphora erythrocephala* (Mg.) et *Lucilia caesar* (L.) (Diptera: Calliphoridae) en metamorphose. Int J Insect Morphol Embryol. 1981 Jan;10(2):173–83. Available from: https://linkinghub.elsevier.com/retrieve/pii/S0020732281800219

[38] Gordya N, Yakovlev A, Kruglikova A, Tulin D, Potolitsina E, Suborova T, et al. Natural antimicrobial peptide complexes in the fighting of antibiotic resistant biofilms: *Calliphora vicina* medicinal maggots. PLoS One. 2017;12(3). doi: 10.1371/journal.pone.0173559 28278280 PMC5344439

[39] Silva MT. When two is better than one: macrophages and neutrophils work in concert in innate immunity as complementary and cooperative partners of a myeloid phagocyte system. Journal of Leukocyte Biology. 2010;87: 93–106. Available from: doi: 10.1189/jlb.0809549 20052802

[40] Sommer C, Koch S, Lammens M, Gabreels-Festen A, Stoll G, Toyka KV. Macrophage clustering as a diagnostic marker in sural nerve biopsies of patients with CIDP. Neurology. 2005;65(12):1924–1929. Available from: doi: 10.1212/01.wnl.0000188879.19900.b7 16380614

